# T Lymphocyte Subset Counts and Interferon-Gamma Production in Adults and Children with COVID-19: A Narrative Review

**DOI:** 10.3390/jpm13050755

**Published:** 2023-04-28

**Authors:** Domenico Umberto De Rose, Pier Giorgio Pace, Francesca Ceccherini-Silberstein, Andrea Dotta, Massimo Andreoni, Loredana Sarmati, Marco Iannetta

**Affiliations:** 1 Neonatal Intensive Care Unit, “Bambino Gesù” Children’s Hospital IRCCS, 00165 Rome, Italy; 2 PhD Course in Microbiology, Immunology, Infectious Diseases, and Transplants (MIMIT), Faculty of Medicine and Surgery, “Tor Vergata” University of Rome, 00133 Rome, Italy; 3 Infectious Disease Unit, Department of System Medicine, “Tor Vergata” University and Hospital, 00133 Rome, Italy; 4 Department of Experimental Medicine, “Tor Vergata” University and Hospital, 00133 Rome, Italy

**Keywords:** SARS-CoV-2, COVID-19 outcome, T cell exhaustion, cytokine storm, inflammation, respiratory outcome, CD3, IFN, IGRA, QuantiFERON-TB Gold, lymphopenia

## Abstract

Adults and children exhibit a broad range of clinical outcomes from SARS-CoV-2 infection, with minimal to mild symptoms, especially in the pediatric age. However, some children present with a severe hyperinflammatory post-infectious complication named multisystem inflammatory syndrome in children (MIS-C), mainly affecting previously healthy subjects. Understanding these differences is still an ongoing challenge, that can lead to new therapeutic strategies and avoid unfavorable outcomes. In this review, we discuss the different roles of T lymphocyte subsets and interferon-γ (IFN-γ) in the immune responses of adults and children. Lymphopenia can influence these responses and represent a good predictor for the outcome, as reported by most authors. The increased IFN-γ response exhibited by children could be the starting point for the activation of a broad response that leads to MIS-C, with a significantly higher risk than in adults, although a single IFN signature has not been identified. Multicenter studies with large cohorts in both age groups are still needed to study SARS-CoV-2 pathogenesis with new tools and to understand how is possible to better modulate immune responses.

## 1. Introduction

Coronavirus disease 2019 (COVID-19) is caused by the seventh coronavirus that is contagious in humans, named severe acute respiratory syndrome coronavirus 2 (SARS-CoV-2). This new β-coronavirus was initially identified in China, and the entire world has been fighting against this pandemic since March 2020.

Lymphocytes play a crucial role in the immune response against viral infections, with lymphocyte activation, undifferentiated lymphocyte proliferation, and a response relying more on T cells and less on antibodies [1]. T and B cell responses after SARS-CoV-2 infection have been observed in the blood roughly one week after the beginning of symptoms [2].

Viral infections can lead to a systematic drop in the number of lymphocytes: lymphopenia can have an influence on the host’s adaptive immune responses and the clinical outcome [3,4,5,6,7]. The reduction in lymphocyte subset counts has proven to be a feasible biomarker for severity stratification in COVID-19 patients in different studies [8,9].

Patients with severe COVID-19 also have impaired interferon (IFN-α, IFN-β, and IFN-γ) production and downregulation of IFN-stimulated genes (ISGs) [10]. A reduced IFN-γ production was found in severely ill COVID-19 patients [11,12].

Individual prognostic factors for COVID-19 remain still unknown. Age is a predictive factor for hospitalization and death, and recent evidence has shown a linear dose–response relationship with mortality [13]. Considering the significant difference in morbidity and mortality between adults and children with COVID-19, the purpose of this review was to highlight recent advances in the understanding of these specific age-related immunological responses to SARS-CoV-2.

## 2. Materials and Methods

This review was produced by searching for articles in the PubMed database and matching the terms “lymphocyte subset” and “interferon response” with “COVID-19” or “SARS-CoV-2”. All retrieved articles written in English and published before 24th March 2022 were analyzed without imposing restrictions on date or year, location, study design, study aims, or inclusion/exclusion criteria. We also screened reference lists of identified studies, and additional references for this review were identified by each author based on their knowledge of the field.

## 3. Differences between Children and Adults with COVID-19

COVID-19 occurs in children of all ages, including newborns [14]; however, pediatric infections represent less than 5% of total cases [15]. In this population, the infection predominates in school children and adolescents in the 6–14-year-old range [16].

Children fare better than adults, with a lower incidence of fever, cough, and fatigue when comparing clinical characteristics among pediatric and adult COVID-19 case series, and implying more asymptomatic infections in children [17]. This pattern is in contrast to what happens for other respiratory viruses, such as in the case of respiratory syncytial virus (RSV), where the prevalence and severity of the infection are higher in children rather than in adults.

According to reports from the United States, fewer children were admitted to hospitals and intensive care units (ICUs) (5.7–20% and 0.58–2.0%, respectively) than adults with an age of 18–64 years (10–33% and 1.4–4.5%, respectively). However, infants had higher hospitalization rates (15–62%) than older children (with ages between 1 and 17 years) (4.1–14%) and adults [17].

In particular, newborn infants can be affected by COVID-19 either indirectly or directly: SARS-CoV-2 infection during pregnancy may be related to an increased risk of preterm delivery or obstetric complications [18,19]. After birth, most neonates present with mild symptoms or are asymptomatic, whereas neonates with severe COVID-19 are more likely to require respiratory support, receive a higher number of medications, and have a longer overall length of stay, especially if they were born preterm [20].

Zimmermann and Curtis summarized that six factors increase the risk of a worse outcome in adults [21]: (1) increased endothelial damage and susceptibility to excessive coagulation with age [22]; (2) age-related differences in the expression of ACE2 receptors and TMPRSS2 cofactor, affinity, and distribution, facilitating SARS-CoV-2 entry into cells [23,24]; (3) pre-existing immunity due to the cumulative exposure to commonly circulating human coronaviruses (229E, HKU1, NL63, OC43) [25]; (4) age-related changes in the immune system, with a decline in innate and adaptive immune function (leading to a lower viral clearance) and chronic proinflammatory state (predisposing to a higher risk of cytokine storm) [26]; (5) comorbidities, such as obesity, diabetes, hypertension, chronic lung, heart and kidney disorders, and smoking [27]; (6) lower vitamin D levels (especially in obesity and chronic kidney disorders), due to its anti-inflammatory and anti-oxidative properties [28]. Conversely, the key factors protecting children comprehend (1) age-related differences in immune response, with a stronger innate immune response and a lower proinflammatory state (protecting from cytokine storm) [29]; (2) more recent vaccination and recurrent viral and *Mycoplasma* infections that can train immunity in clearing SARS-CoV-2 [30]; (3) differences in nasopharyngeal and gastrointestinal microbiota [31,32]; (4) higher melatonin levels, which have anti-inflammatory and anti-oxidative properties [33]; (5) lower workplace, shopping, travel, and nosocomial exposure to SARS-CoV-2, with consequent lower initial viral load [21,34].

However, in a cross-sectional study of 555 children and adults with SARS-CoV-2 confirmed by reverse transcription polymerase chain reaction, symptomatic individuals had higher SARS-CoV-2 RNA levels (as indicated by lower mean cycle threshold values) compared with asymptomatic individuals. No significant differences in RNA levels were found between asymptomatic children and asymptomatic adults or between symptomatic children and symptomatic adults [35].

Furthermore, emerging evidence shows that both adults and children may develop post-acute sequelae of SARS-CoV-2 infection: children seem to experience persistent multisystemic symptoms months after diagnosis of mild acute SARS-CoV-2 infection, although less frequently and less severely than co-habitant adults [36].

## 4. T Lymphocyte Subset Counts: Current Knowledge and Clinical Implications

### 4.1. T-Lymphocyte Subset Counts in Adults

Huang and Pranata showed in a meta-analysis that lymphopenia at hospital admission was associated with poor outcomes in patients with COVID-19 [37], but there is increasing evidence focusing on the role of specific lymphocyte subset counts in predicting outcomes [2,37,38]. Moreover, lymphopenia at admission, when associated with neutrophilia, has been associated with a worse outcome [39]: Liu et al. described that the lower the T cells, and in particular, CD4+ and CD8+ T cell counts, the more serious the disease and the worse the prognosis [40]. Diao et al. delineated that hospitalized patients with a total T cell count of less than 800 µL may require urgent interventions, even if severe symptoms are initially absent, due to a high risk of further deterioration in their general conditions [41].

All T cell subsets were significantly decreased in non-survivors [42], but a global reduction in the absolute number of CD4+ T cells is common among COVID-19 patients (especially those with severe manifestations) with different molecular and cytokine profiles [43]. Both older age and lower CD4+ T cells count, indicators of immunosuppression, were significantly associated with intensive care unit (ICU) admission, according to Chen’s findings [44]. Iannetta et al. described that total CD3+ T lymphocyte and CD3+CD4+ subset absolute counts were significantly related to in-hospital mortality, together with older age, male gender, increased LDH, and creatinine plasmatic levels [9]. Indeed, CD4+ T cells have a direct and indirect role in the immune response to SARS-CoV-2 infection, coordinating antiviral responses and interfering with viral replication [38].

Similarly, CD8+ T cells are involved in antiviral response: a CD8+ T cell clonal expansion either in bronchoalveolar lavage fluid or peripheral blood has been defined as being directly related to COVID-19 recovery or milder symptoms of the disease [45]. In particular, according to Du’s findings, CD3+CD8+ T cells ≤ 75 cells/μL, associated to age ≥ 65 years, pre-existing concurrent cardiovascular or cerebrovascular disorders, and cardiac troponin I ≥ 0.05 ng/mL, were significantly associated with increased risk of death in patients with pneumonia caused by SARS-CoV-2 [46].

The ratio between CD4+ and CD8+ T cells is typically considered normal when it is between 1.5 and 2.5 [47]. According to our data, we found no differences in the CD4+/CD8+ ratio between survivors and non-survivors, probably due to the equal reduction in CD4+ and CD8+ T lymphocytes [9]. Similarly, Wang et al. described a group of patients with COVID-19 pneumonia who had significantly lower CD4+ T cells and CD8+ T cells and no significant difference in the CD4+ to CD8+ ratio [48]. Conversely, a recent meta-analysis including 145 studies, and, thus, in a larger sample, identified that a higher ratio of CD4+ to CD8+ T cells was associated with a fatal outcome [49].

Furthermore, our group calculated a “T Lymphocyte subset index” (TLSI) as the number of T lymphocyte subset absolute counts below the cut-off value, ranging from 0 to 4 (all subpopulation absolute counts above or below the cut-offs, respectively). It was revealed to be an independent predictor for in-hospital mortality and disease severity in COVID-19 hospitalized patients and can represent a useful index with the ability to summarize alterations of different T lymphocyte subsets, indicating that a higher value of the index is associated with an increased risk of severe disease (OR 2.60 (CI 95%: 1.66–4.06)) and in-hospital mortality (OR 2.18 (CI 95%: 1.00–4.76)) [9].

Beyond the absolute number of T cells, Meckiff et al. showed that hospitalization is associated with increased cytotoxic follicular helper cells (TFH) and cytotoxic T helper cells (CD4-CTLs) and a reduction in regulatory T cells (TREGs) [50]. Sadeghi et al. confirmed that the number of circulating TREG cells was meaningfully reduced in COVID-19 patients admitted to the ICU. They found a higher ratio of TH17/TREG cells in COVID-19-dead patients compared with cases that gradually improved, with a strong relationship between this response and related cytokines with inflammation and mortality [51].

Recently the results of a multi-omics study (COVID-19 Multi-Omic Blood Atlas, COMBAT Consortium) highlighted an increased frequency of activated CD4+ and CD8+ T cells in all COVID-19 patient groups, with a reduction in CD4+ TH1, CCL5+CD8+ T central memory, CD45RA+CD8+ T effector memory, and NK cells in hospitalized cases. They also remarked on the evidence of change in innate-like lymphocytic cell populations with increasing COVID-19 severity, including mucosal-associated invariant T (MAIT) cells, with a higher percentage of CD69+ MAIT cells in cases of severe clinical presentation [52]. Qin et al. indicated a reduction in the number of memory TH cells (CD3+CD4+CD45RO+), which can explain why COVID-19 patients could not, at least in some cases, develop a definitive immunity against future infection with the virus [53].

Analyzing CD4+ T cells that are reactive against the spike glycoprotein of SARS-CoV-2 in the peripheral blood, Braun et al. detected spike-reactive CD4+ T cells not only in 83% of patients with COVID-19 but also in 35% of SARS-CoV-2-unexposed healthy donors. This is related to the cross-reactivity of these T cells with spike protein C-terminal epitopes of human endemic coronaviruses 229E and OC43, which were most likely generated during previous encounters with these viruses [54].

**Figure 1:**
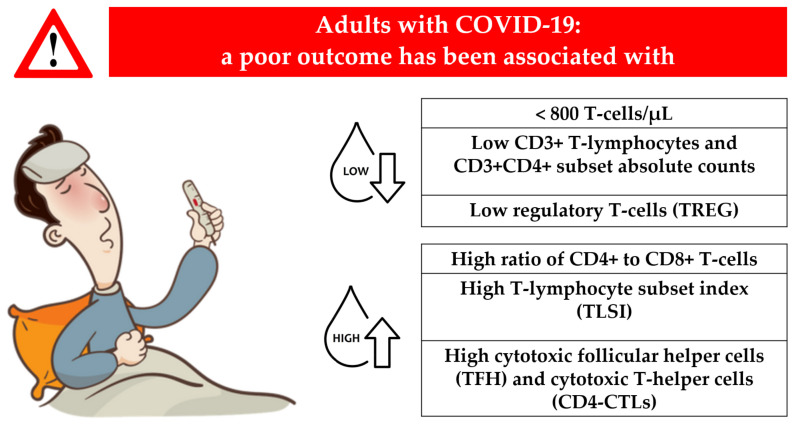
Main findings associated with a poor outcome in adults with COVID-19.

The main findings associated with a poor outcome in adults with COVID-19 are summarized in Figure 1.

Finally, there are an increasing number of works in the literature about “Long COVID”, a term that refers to signs and symptoms that persist after an acute SARS-CoV-2 infection, although the knowledge about this condition is still limited, probably due to the variability of definitions used in different studies [55]. Considering that the reappearance of effector T cells is associated with recovery from COVID-19 [56], it is interesting to note that patients with long COVID had decreased CD4+ and CD8+ effector memory (EM) cell numbers and increased programmed cell death protein 1 (PD-1) expression on central memory (CM) cells [57].

### 4.2. T Lymphocyte Subset Counts in Children

Children suffering from COVID-19 usually have milder symptoms and a better prognosis than adults [58], and there are fewer studies about T lymphocyte subset counts in children than in adults [59,60,61]. In contrast to acute pediatric COVID-19 infection, the multisystem inflammatory syndrome in children (MIS-C) appears to be a more severe condition (Table 1), with myocardial dysfunction and coronary artery dilation/aneurysms, and 68% of cases requiring critical care support [62].

MIS-C is a multiorgan hyperinflammatory disease that mimics some of the Kawasaki disease features. Children with MIS-C are usually healthy and without concomitant diseases. Both innate and adaptive immune cells participate in various immune pathways leading to systemic inflammation, immune overactivation, and antibody-mediated tissue destruction. Moreover, genetic predisposition plays a crucial role [63].

The epidemiology of MIS-C is still unclear, although it appears to be a relatively rare condition, with an incidence lower than 1% in SARS-CoV-2-infected children. As in children, adults who have been infected with SARS-CoV-2 can develop MIS (MIS-A) days to weeks after becoming sick with COVID-19, but this condition is even rarer in adults [64]. A growing body of work in the literature focuses on the immunological profiles of pediatric COVID-19 and MIS-C.

With increasing disease severity, the T lymphocyte subset and natural killer cell numbers also declined in pediatric patients, according to Lu’s findings. Critical patients showed an increased CD4+/CD8+ ratio compared to subjects with milder presentations [60], as in adults [49].

Conversely, Jia et al. found no significant changes in CD3+, CD3+CD4+, and CD3+CD8+ in the acute phase, whereas they evidenced a statistically significant transient increase in T helper 2 (TH2) cells, indicating an early TH2-dominant immune response to the viral infection. Furthermore, regulatory T cells (TREGs), which have an immunosuppressive function, appeared to be most likely suppressed in patients during the acute phase, returning to normal levels in the convalescence phase, as were TH2 cells [65].

Interestingly, Li et al. revealed that children with SARS-CoV-2 pneumonia have a greater count of CD3+CD8+ lymphocytes and a larger proportion of CD3+ and CD3+CD8+ lymphocytes when compared to children with pneumonia due to respiratory syncytial virus (RSV) (a well-known infection in pediatrics), without significant differences between the two groups in terms of CD4+/CD8+ ratio, CD3+, CD3+CD4+, and CD16+CD56+ lymphocyte counts, or the proportion of CD3+CD4+ and CD16+CD56+ cells [61].

**Figure 2:**
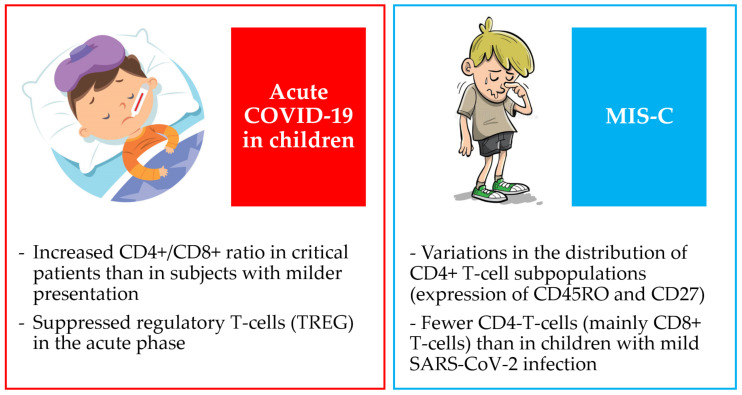
Main findings about T cell subsets in children with COVID-19 and MIS-C.

To better clarify the hyperinflammation in MIS-C and distinguish it from Kawasaki disease, Consiglio et al. performed a flow cytometric analysis of peripheral blood mononuclear cells (PBMC) obtained from children at hospital admission. They revealed variations in the distributions of CD4+ T cell subpopulations identified by the expression of CD45RO and CD27, as well as the frequency of T-follicular helper cells (TFH) expressing the chemokine receptor CXCR5. In comparison to children without Kawasaki disease or MIS-C, total T cell counts were lower in both forms of hyperinflammation [66]. MIS-C patients and children with mild SARS-CoV-2 infection exhibited comparable subset distributions within the CD4+ T cell compartment, showing that changes found in comparison to healthy children may be linked to the SARS-CoV-2 infection itself. Furthermore, children with MIS-C had considerably fewer CD4- T cells (mainly CD8+ T cells) than children with mild SARS-CoV-2 infection (Figure 2) [66].

In particular, in 75% of patients with MIS-C, but not in any patient with toxic shock syndrome, Kawasaki disease, or acute COVID-19, Moreews et al. noted a distinct polyclonal expansion of activated T cells expressing the V21.3 T cell receptor chain variable region, which can be considered a hallmark of MIS-C [67].

Since plasmatic dendritic cells induce naïve T cell activation, Badolato’s group also investigated dendritic cell subsets, finding low levels in the peripheral blood of MIS-C patients, as compared to COVID-19 children [68].

Evaluating the presence of immunological memory T cells in the peripheral blood of children who had recovered from mild COVID-19, Tian et al. demonstrated that in children aged 0 to 4 years, both CD4+ and CD8+ T cell levels exhibited a considerable rise in central memory (CD45RA- CCR7+ (TCM)) and effector memory (CD45RA- CCR7+ (TEM)) cell proportions. However, no significant variations in memory cell levels were detected in the 5-to-9-year-old groups. Moreover, in the 10-to-14-year-old group, the frequencies of effector memory CD45RA+ (CD45RA+ CCR7 (TEMRA)) cells among CD4+ cells and TCM cells among CD8+ cells increased. Furthermore, no differences in the percentage of naïve T (CD45RA+ CD27+ CCR7+) and memory T cell subtypes were seen in children of various ages who recovered from mild COVID-19 [69]. The same authors suggested that memory T cells specific for SARS-CoV-2 S and N protein were produced and maintained in the peripheral blood of COVID-19 convalescent children [69].

## 5. IFN-γ Production: Current Knowledge and Clinical Implications

### 5.1. Basal IFN-γ Production in Adults

The results of studies about IFN-γ production are controversial, with levels found in unstimulated plasma that more reflect the number and activation of NK cells (which intervene rapidly as an innate response) than the response of T lymphocytes (which intervene later as an adaptive response). However, SARS-CoV-2 infection may predominantly affect T lymphocytes, notably CD4+ and CD8+ T cells, and this, accordingly, is related to a lower IFN-γ production due to reduced CD4+ T cell counts. In particular, IFN-γ expression by CD4+ T cells was lower in severe cases (14.1%) than in moderate cases (22.8%), according to Chen et al. [70]. Considering that IFN-γ inhibits the TH2 pathway [71], the dominant TH2 response in COVID-19 patients could be explained by the low IFN-γ levels [72].

In addition, Diao et al. found no differences in serum levels of IFN-γ between patients admitted to the intensive care unit (ICU) and those with a milder presentation. However, they described how serum levels of IFN-γ were significantly lower in COVID-19 patients in the recovery rather than in the acute period: the differences in these levels can be related to the time of blood sampling, in addition to the severity of included patients [41].

Gadotti et al. confirmed that IFN-γ was higher in the early stages of the disease [72]. Objective risk stratification should include factors that can be easily obtainable and reproducible to better compare results from different studies.

According to post-mortem studies revealing a mononuclear cell increase in the lungs [73], the absence of change in IFN-γ plasmatic levels may suggest a stronger response in lymphoid organs and sick tissues, which may, therefore, fail to be observed during peripheral blood cytokine evaluation [74].

Grant et al. described circuits between infected macrophages and T cells in SARS-CoV-2 pneumonia. Memory T cell activation results in IFN-γ production, local proliferation of activated memory T cells, activation of inflammatory responses, and recruitment of monocytes and T cells. They found that patients with SARS-CoV-2 pneumonia had more CD4+ and CD8+ T cells in their alveolar area than patients with other infections. Furthermore, their single-cell RNA-sequencing data on bronchoalveolar fluid collected less than 48 h after intubation showed that both CD4+ and CD8+ T cells produce IFN-γ [75].

While innate immune cells generate a variety of inflammatory cytokines in response to the SARS-CoV-2 infection, Karki et al. discovered that only the combination of TNF-α and IFN-γ caused inflammatory cell death characterized by PANoptosis (pyroptosis, apoptosis, and necroptosis). When treating mice with neutralizing antibodies against TNF-α and IFN-γ, they prevented mice from contracting SARS-CoV-2 infection, sepsis, hemophagocytic lymphohistiocytosis, or cytokine shock [76].

Considering that IFN-γ triggers antiviral and adaptive immune responses through a JAK/STAT signaling pathway, JAK inhibitors have been used in COVID-19 patients. Among them, baricitinib has obtained positive results in clinical trials, reducing mortality and the need for mechanical ventilation in patients hospitalized for COVID-19 [77,78].

### 5.2. IFN-γ Production after Stimulation in Adults

Imeneo et al. used the QuantiFERON-TB Gold Plus (QFT-Plus) test (Qiagen, Germany), an interferon-γ release assay (IGRA), to detect the secretion of IFN-γ produced by lymphocytes specifically sensitized by *Mycobacterium tuberculosis* antigens and aspecifically by phytohemagglutinin (PHA) ex vivo: they found that in patients with severe COVID-19, the profound decrease in the peripheral blood T cell subsets (CD3+, CD4+, and CD8+) was related to an increase in QFT-Plus indeterminate rates, thus, reflecting a reduced IFN-γ production [12]. The rates of the indeterminate QFT-Plus assay were higher, especially in patients requiring non-invasive or invasive ventilation rather than in those treated only with a Venturi mask or not needing oxygen supplementation [12]. Ward et al. equally found, using the same method, that their COVID-19 patients had a six-fold decrease, on average, in their ability to produce IFN-γ compared to healthy controls [11]. Indeed, Ni et al. used IFN-γ ELISpot (MABTECH AB, Sweden), confirming a trend towards a reduction in T cells (in particular CD8+) with a related decrease in IFN-γ production after stimulation with recombinant SARS-CoV-2 proteins [79].

Conversely, Petrone et al. illustrated that the whole-blood IFN-γ response to SARS-CoV-2 peptides was detected independently of the disease severity (mild or moderate or severe/critical disease), symptoms onset (within 15 days, 16–30 days or more than 31 days), and lymphocyte counts (<1000/μL; ≥1000/μL and <2000/μL; >2000/μL) [80].

According to Lucas’s findings, always following stimulation, plasma levels of interferon, including IFN-γ, are correlated with nasopharyngeal viral load, regardless of disease severity [81]. Similarly, Tincati et al. also showed similar plasma IFN-γ levels between mild and severe COVID-19 patients; however, they found that the frequencies of CD4+ T cells producing IL-4 and IFN-γ were lower than expected after stimulation with a peptide pool [82].

### 5.3. IFN-γ Production in Children

**Figure 3:**
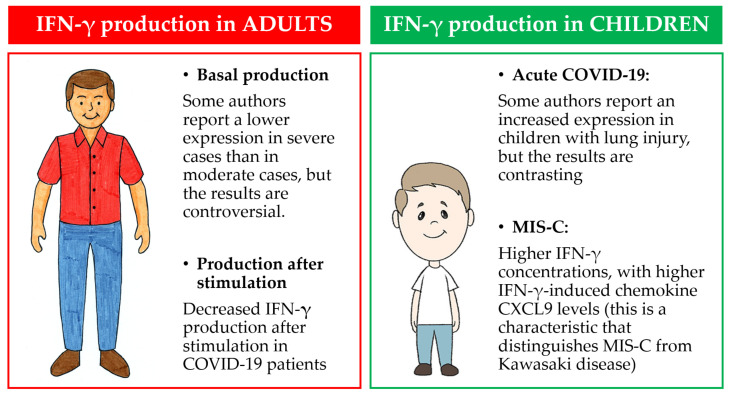
Main findings about IFN-γ production in adults and children.

Few data are available about interferon signatures for pediatric patients compared to adult ones, and the results are contrasting (Figure 3).

For the first time, Sun et al. described increased plasma concentrations of pro-inflammatory cytokines, including IFN-γ, in a few children with COVID-19 lung injury from Wuhan (China) [83].

Shafiek et al. found no significant differences in IFN-γ production in a multicenter Egyptian study enrolling 92 symptomatic children with COVID-19 pneumonia and 100 healthy controls [84]. Conversely, Tovo et al. in Italy enrolled 64 children with mild/moderate or severe clinical manifestations of COVID-19 and 60 uninfected children as controls, assessing the transcriptional levels of IFN-γ and its ISGs. They demonstrated that IFN-γ and its specific ISGs (chemokine C-X-C motif ligand 10 —CXCL10— and Indoleamine 2,3-Dioxygenase 1 —IDO1—) are more expressed in patients with mild COVID-19 presentation, but they tend to decrease in children with severe clinical manifestations, particularly in MIS-C [85].

However, a potential role for IFN-γ in MIS-C pathogenesis has been suggested in a multicenter cohort reported by Hoste et al., with significantly elevated levels of CXCL9 (chemokine C-X-C motif ligand 9) and CXCL10 in both MIS-C and COVID-19 patients [86], in line with similar observations in MIS-C by Caldarale et al. (in Italy) [68], Carter et al. (in the United Kingdom) [87], Esteve-Sole et al. (in Spain) [88], and Diorio et al. (in the United States) [89].

Rodriguez-Smith’s data reveal that MIS-C can be distinguished from Kawasaki disease predominantly by higher IFN-γ-induced chemokine CXCL9 levels: the CXCL9 concentration has also been documented as related to disease severity [90].

Similarly, Pierce et al. described higher IFN-γ concentrations in pediatric patients with MIS-C from the United States rather than in adults (both adults who recovered and did not require mechanical ventilation and adults who required mechanical ventilation or died), as well as in comparison to children with acute COVID-19 not requiring respiratory support [29]. They speculated that the higher concentrations of IFN-γ in children’s serum reflected an increased production of innate immune cells in the respiratory tract, where local cytokines may have protected them from a lung injury, but they initially did not investigate levels in respiratory samples. A subsequent study provided direct evidence of higher levels of IFN-γ (and other mediators of innate pathways) in nasal fluid in children than in adults [91].

Another multicenter study confirmed that MIS-C is characterized by transient acute proinflammatory hypercytokinemia, including elevated levels of IFN-γ and interleukins 6, 10, and 17A, in addition to specific antibodies; these cytokines were significantly associated with a prolonged duration of hospitalization, but not to other categorical outcomes (intensive care unit admission, impaired cardiac function, shock, pneumonia). In convalescence, cytokine levels dropped, and antibody titers waned, indicating transient immune activation and hyperinflammation in MIS-C responses [92].

Phospholipases participate in host inflammatory responses and are linked to the release of damage-associated molecular pattern molecules (DAMPs), which activate the innate immune response. Beyond dysregulated IFN-γ responses, the phospholipase PLA2G2A was significantly higher in SARS-CoV-2-infected pediatric patients than in healthy ones, with the most marked difference in those with MIS-C. It seems to be associated with a thrombotic microangiopathy phenotype, with vascular or platelet dysfunction surrogate biomarkers. Interestingly, PLA2G2A levels do not improve over time throughout convalescence [89].

Another interesting feature was the low expression of genes involved in IFN-I and IFN-II responses, especially in monocytes and dendritic cells of children with severe myocarditis, if IFN-γ and IFN-α2 proteins were elevated in the plasma of all individuals with MIS-C [93].

Patients with MIS-C are mostly children older than 7 years [94], but there are an increasing number of works in the literature describing neonates born to mothers with SARS-CoV-2 infection (MIS-N) and infants infected with SARS-CoV-2 who presented with severe disease (MIS-C) [95]. In young infants under 3 months old, Goenka et al. described that strong functional antibody responses (high anti-spike IgG levels in sera and saliva) combined with tempered IFN-γ production may effectively protect infants from severe COVID-19 complications [96].

Different limitations of these studies must be considered: measurements are assessed in small samples of pediatric patients with different clinical manifestations (mild/moderate COVID-19, severe COVID-19, MIS-C), and treatments with steroids, immunoglobulins, and other drugs that could have influenced the results are not always reported.

Moreover, genetic factors can influence the pediatric vulnerability to MIS-C: an immune impairment emerged in 3/18 patients with MIS-C (16.7%), in whom a whole-exome sequencing (WES) was performed. In particular, the haploinsufficiency of the suppressor of cytokine signaling 1 (SOCS1), a negative regulator of type I and II interferon pathways, has been identified as a genetic risk factor for MIS-C [97]. Further studies are needed in order to detect potential inborn errors of immunity in patients with MIS-C through clinical WES.

## 6. Limitations

The limitations of most studies that we identified are their retrospective nature and the single-center design for both T lymphocyte subset counts and IFN-γ production. Furthermore, available data are nonhomogeneous, with different clinical criteria for severity when including patients in each group (milder forms versus severe disease), the timing of inclusion and blood sampling, and the different outcomes that were evaluated. This still prevents us from drawing conclusions, although the identification of a distinctive signature in T lymphocyte cells could help to identify and rapidly intercept patients with a higher risk of clinical deterioration.

While enrolling SARS-CoV-2 infected patients with acute infections is not difficult, the incidence of MIS-C is extremely low (while MIS-A is even more so), leading to the necessity to design prospective multicenter studies to reach significant samples.

Furthermore, the role of IFN-γ should be explored not only in the acute phase of COVID-19 but also in the recovery and the presence of a long COVID spectrum, both in adults and in children. An already validated method to quantify IFN-γ production after stimulation (rather than dosing the basal levels only), such as interferon-γ release assays, should be used to allow data comparisons between different centers.

## 7. Conclusions and Future Directions

We demonstrated that there is emerging evidence in COVID-19 patients about the changes in T lymphocyte subset counts and IFN-γ production, which are crucial for escalating antiviral responses. In order to better compare results from different centers, future studies should identify mild COVID-19 patients and moderate to severe COVID-19 patients, always using the same clinical criteria.

Indeed, further studies are needed to confirm these changes and understand age-related differences that can explain variations in morbidity and mortality between adult and pediatric populations. This can be the starting point for identifying targeted treatments and modifying the natural course of the viral infection, especially when comorbidities are present.

## Figures and Tables

**Table 1:** Clinical and laboratory findings in children with MIS-C.

	MIS-C Clinical Findings
Age	More frequent in older children (>8 years old)
Sex	More frequent in males
Fever	Yes (about 3–5 days)
Cardiovascular involvement	Myocardial dysfunction, coronary artery dilation/aneurysms, arrhythmias
Respiratory involvement	Tachypnea, acute respiratory failure requiring noninvasive or invasive ventilation
Gastrointestinal involvement	Abdominal pain, vomiting, diarrhea
Neurological involvement	Headache, lethargy, confusion, irritability
Skin involvement	Rash, red or swollen lips, strawberry tongue
Renal involvement	Acute kidney injury
Other clinical findings	- Serositis (small pleural, pericardial, and ascitic effusions) - Hepatitis or hepatomegaly - Possible lymphadenopathy
Laboratory anomalies	- Lymphocyte count: normal (1000–4800/μL) to low (<1000/μL) - Neutrophil count: normal (1800–7800/μL) to high (>10,000/μL) - Platelets: normal (150–450,000/μL) to low (<150,000/μL) - Liver function tests: normal to high (>2–5 upper limit of the normal) - Triglycerides: normal (<90 mg/dL) - Fibrinogen: normal (150–400 mg/dL) to low (<150 mg/dL) - Ferritin: normal (15–300 ng/mL) to high (>300 ng/mL) - C-reactive protein: high (>3.0 mg/dL)

## Data Availability

Not applicable.
